# Rheological Properties and 3D Printing Behavior of PCL and DMSO_2_ Composites for Bio-Scaffold

**DOI:** 10.3390/ma17102459

**Published:** 2024-05-20

**Authors:** Jae-Won Jang, Kyung-Eun Min, Cheolhee Kim, Chien Wern, Sung Yi

**Affiliations:** 1 Department of Mechanical and Material Engineering, Portland State University, Portland, OR 97201, USA; jaewon@pdx.edu (J.-W.J.); min3@pdx.edu (K.-E.M.); cheol@pdx.edu (C.K.); cwern@pdx.edu (C.W.); 2 Welding and Joining R&D Group, Korea Institute of Industrial Technology, 156, Getbeol-ro, Yeonsu-gu, Incheon 21999, Republic of Korea

**Keywords:** PCL, DMSO_2_, rheology, viscosity, 3D printing behavior, biomaterial, scaffold

## Abstract

The significance of rheology in the context of bio three-dimensional (3D) printing lies in its impact on the printing behavior, which shapes material flow and the layer-by-layer stacking process. The objective of this study is to evaluate the rheological and printing behaviors of polycaprolactone (PCL) and dimethyl sulfone (DMSO_2_) composites. The rheological properties were examined using a rotational rheometer, employing a frequency sweep test. Simultaneously, the printing behavior was investigated using a material extrusion 3D printer, encompassing varying printing temperatures and pressures. Across the temperature range of 120–140 °C, both PCL and PCL/DMSO_2_ composites demonstrated liquid-like behavior, with a higher loss modulus than storage modulus. This behavior exhibited shear-thinning characteristics. The addition of DMSO_2_ 10, 20, and 30 wt% into the PCL matrix reduced a zero-shear viscosity of 33, 46, and 74% compared to PCL, respectively. The materials exhibited extrusion velocities spanning from 0.0850 to 6.58 mm/s, with velocity being governed by the reciprocal of viscosity. A significant alteration in viscosity by temperature change directly led to a pronounced fluctuation in extrusion velocity. Extrusion velocities below 0.21 mm/s led to the production of unstable printed lines. The presence of distinct viscosities altered extrusion velocity, flow rate, and strut diameter. This phenomenon allowed the categorization of pore shape into three zones: irregular, normal, and no-pore zones. It underscored the importance of comprehending the rheological aspects of biomaterials in enhancing the overall quality of bio-scaffolds during the 3D printing process.

## 1. Introduction

In tissue engineering applications, the three-dimensional (3D) printing method holds great promise for creating scaffolds that mimic the extracellular matrix of tissues, can support the growth of cells, and promote tissue regeneration [1]. As the scaffold is fabricated with biocompatible materials, these scaffolds provide a framework for cells to adhere, increase, and differentiate, enabling the regeneration of damaged or lost tissues [2]. The 3D printing method has been employed for 3D bio-scaffold manufacturing due to accurate and repeatable fabrication abilities [3]. Material extrusion 3D printing technology is a popular additive manufacturing technology used in tissue engineering [4]. In this technique, a 3D printer extrudes and deposits a thermoplastic material in a molten state through a nozzle, layer by layer, to create a 3D scaffold or structure [5]. Material extrusion 3D printing offers precise control over scaffold design and has shown promising applications in various tissue engineering applications, including cartilage [6], bone [7], skin [8], and vascular tissues [9].

Rheology examines how materials flow and deform under stress and plays a crucial role in 3D printing. Although numerous critical parameters such as the printing temperature of the nozzle, print bed condition, chamber environment, nozzle speed, and layer thickness need to be considered, particularly for thermoplastic polymer 3D printing [10,11], the rheological properties of the material directly impact printability, layer adhesion, mechanical properties, and the overall quality of the produced 3D objects [12,13,14]. The material extrusion process, especially printability, relies heavily on rheology. In material extrusion 3D printing, the biomaterial, mainly thermoplastic materials, are extruded through a nozzle, building the object layer by layer. The viscosity and shear thinning behavior of the material affect its flow through the nozzle, directly influencing the precision and accuracy of the printed structure [15]. In addition, rheological properties can determine the strength of layer adhesion and impact the extrusion velocity, affecting the overall quality of 3D-printed objects [16]. Materials with higher viscosities may require slower print speeds to maintain accuracy, while low-viscosity materials can be printed faster [17]. Rheology plays a vital role in successfully implementing 3D printing, impacting the material selection, printing process, and final properties of the printed objects.

Polycaprolactone (PCL) has emerged as a popular biodegradable material for 3D bio-scaffold fabrication in 3D printing [18]. This semi-crystalline polyester exhibits high ductility and biocompatibility. Its production involves ring-opening polymerization of ester monomers, resulting in a wide range of molecular weights. Several researchers have directed their attention to the rheological properties of PCL for its application in 3D printing [19,20,21]. Noroozi et al. [22] examined the rheological behavior of PCL in correlation with its molecular characteristics, aiming to understand its flow behavior. They observed a positive relationship between the zero-shear viscosity of PCL and its molecular weight. On the other hand, Arraiza et al. [23] focused on the thermal degradation of PCL, focusing on its rheological properties. Their investigation revealed that PCL displayed a pseudoplastic behavior, and its viscosity adhered well to the power law.

Despite being extensively studied for tissue engineering applications, PCL has certain drawbacks, such as low mechanical properties, including elastic modulus and yield strength, as well as a long degradation time. To overcome these limitations, researchers have studied PCL-based composites. Luiz et al. [24] incorporated cellulose nanocrystals as a mechanical reinforcement material in PCL. Their study revealed a significant increase of 155% in the elastic modulus with 25 wt% of cellulose nanocrystals compared to pure PCL. Doyle et al. [25] aimed to modulate the degradability of PCL by adding nanohydroxyapatite to create a composite. The composite containing 30% nanohydroxyapatite demonstrated the fastest weight loss after 72 h in an accelerated degradation test using 0.1 M NaOH. However, the addition of nanohydroxyapatite led to an increase in viscosity, causing difficulties in material flow. Similarly, Dimethyl sulfone (DMSO_2_) was introduced as a reinforcement material in PCL [26,27]. PCL and DMSO_2_ composites exhibited improved mechanical properties, reduced water contact angle, and faster mass loss. However, the rheological and printing behavior of these composites has not been reported yet.

The objective of this study is to evaluate the rheological and printing behavior of PCL and PCL-based composites, which incorporate 10, 20, and 30 wt% of DMSO_2_. Rheological behavior was investigated using a rotational rheometer, specifically focusing on selected temperatures above the melting temperature of DMSO_2_. Additionally, the printing behavior was analyzed using a material extrusion-based 3D printer, considering various temperatures and pressures. The present study seeks to enhance our understanding of the intricate relationship between these materials’ rheological properties and printing behavior.

## 2. Experimental

### 2.1. Materials

In this study, two materials were utilized, and four composites were prepared. A white-colored powdered PCL was obtained from Polysciences (Warrington, PA, USA), while the powdered DMSO_2_ of the same color was provided by Bergstrom Nutrition (Vancouver, WA, USA). The material properties supplied by the manufacturers are presented in Table 1. The concentrations of DMSO_2_ in the PCL matrix, namely 10, 20, and 30 wt%, were prepared based on references [26]. Prior to the mixing process, both PCL and DMSO_2_ were dried under vacuum conditions at 45 °C for one day. A physical mixing procedure was conducted using an electric milling machine (YUESUO, Zhengzhou, China). As in the previous study [26], the composites in this study were designated as PCL/D10, PCL/D20, and PCL/D30, indicating their respective DMSO_2_ weight percentages.

### 2.2. Rheological Measurements

Polymers exhibit both viscous and elastic behavior, and their viscoelastic response is often described by the complex shear modulus (G*), which combines both components. Viscoelasticity in polymers is often characterized by time and frequency dependency. The frequency sweep test can measure the complex shear modulus, including storage and loss modulus and phase angle. The complex shear modulus can be expressed as follows:

(1) $$G^{*}=G^{\prime }+iG^{\prime \prime },$$

where *G*′ is the storage modulus and *G*″ is the loss modulus. Then, the complex viscosity of polymer ($\eta^{*}$) can be calculated by the complex shear modulus as follows:

(2) $$\eta^{*}=\frac{\sqrt{G^{\prime 2}+G^{\prime \prime 2}}}{\omega },$$

where ω is the frequency.

In this study, the rheological properties of PCL and its composites were determined using an MCR 702 rotational rheometer (Anton Paar, Graz, Austria) equipped with a parallel plate system featuring a 0.5 mm gap to determine the zero-shear viscosity from the complex shear modulus and the complex viscosity. Frequency sweep tests were performed after preheating for 180 s at temperatures of 120, 130, and 140 °C, with a strain of 1% and frequencies ranging from 0.1 to 102.4 rad/s. The measuring points were divided into 11 points across the frequency range on a logarithmic scale.

### 2.3. 3D Printing Process and Measurement of the Extrusion Velocity and Strut Diameter

A pneumatic material extrusion 3D printer using compressed air, Allevi 2 (Allevi, Inc., Philadelphia, PA, USA), was utilized for material printing and sample preparation. This 3D printer can set a temperature and pressure ranging from room temperature to 140 °C and 6.90–827 kPa, respectively. A full metal nozzle with an inner diameter of 450 µm was attached, and the nozzle moving speed and the print bed temperature were fixed at 0.8 mm/s and ambient room temperature, individually, in this study. The extrusion of the materials from the nozzle was captured by an EOS REBEL T3i DSLR camera (CANON, Tokyo, Japan), and the captured videos were converted into images at a rate of 30 frames per second to calculate the extrusion velocity of the materials. The extruded materials were stacked on the substrate with a length of 2 mm and layer height of 450 µm. The layer height was set the same as the nozzle inner diameter. The printed strut on the substrate was employed to measure the strut diameter.

The cylindrical sample having open pore (target porosity of 55–58%) was designed to investigate the pore generation using SOLIDWORKS 2021 (Dassault Systèmes, Vélizy-Villacoublay, France), and the STL file was generated. The designed 3D object was sliced using open source software Slic3r (version 1.2.9), and the created G-code file was used for 3D printing. The printing process was carried out at the same ranges of the printing temperature and pressure with the line printing conditions, resulting in samples with a diameter of 25 mm and a height of 1 mm. The first layer’s thickness was set as 315 µm (70% of the nozzle inner diameter), and the other layers’ thickness was set as 270 µm. A total of five layers were printed for the cylindrical 3D object.

All measurements, including rheology, extrusion velocity, and strut diameter, were performed five times per material. This study used Mean values as representative values, and error bars were not presented in the results. The error values were negligible, being less than 0.5%, and thus considered insignificant.

## 3. Results and Discussion

### 3.1. Rheological Properties

In the material extrusion 3D printing method, the rheological properties of biomaterial are a significantly important parameter for the extrusion and flow process [28]. The storage modulus and loss modulus were measured as a function of the frequency in the range suggested in the experimental part and the shear viscosity.

**Figure 1:**
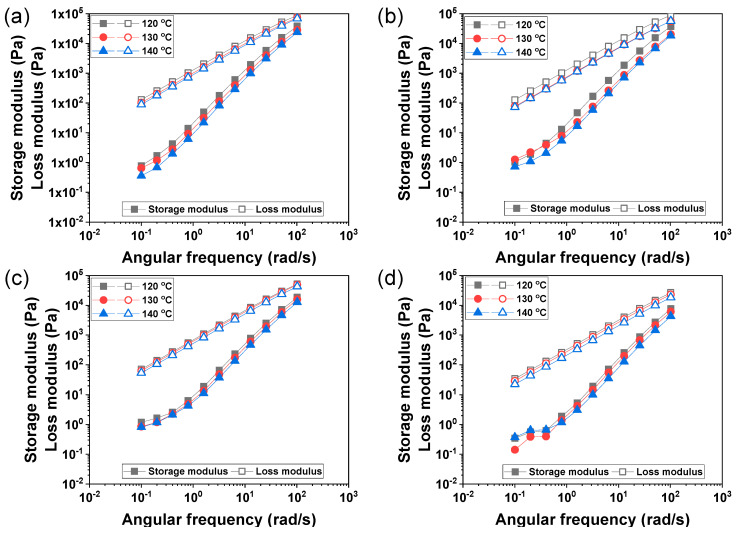
Storage modulus (solid symbol) and loss modulus (open symbol) of (**a**) PCL, (**b**) PCL/D10, (**c**) PCL/D20, and (**d**) PCL/D30 at 120, 130, and 140 °C.

A liquid-like behavior dominated at the selected angular frequency and temperature for pure PCL and PCL-based composites, indicating that the loss modulus was over the storage modulus (Figure 1). Moreover, the composites’ modulus decreased as the DMSO_2_ contents were increased in the PCL matrix. This phenomenon, similar to decreasing the modulus of the composite in the binary system, has been reported for nano-sized particle addition into the matrix [29].

When low-viscosity material is mixed with high-viscosity material, the composites can exhibit a dilution effect. The viscosity of molten PCL with a molecular weight of 50,000 at 100 °C is known to be about 1670 Pa·s [22], while the viscosity of liquid DMSO_2_ at 123 °C is known to be about 1.14 mPa·s [30]. The viscosity of molten PCL is significantly higher than that of liquid DMSO_2_. This substantial viscosity difference can lead to a significant dilution effect in PCL and DMSO_2_ composites. Moreover, the dilution effect can lead to a decrease in the modulus of the composites. Moreover, the rheological behavior of the composites demonstrated a similar trend to that observed in the behavior of the modulus.

**Figure 2:**
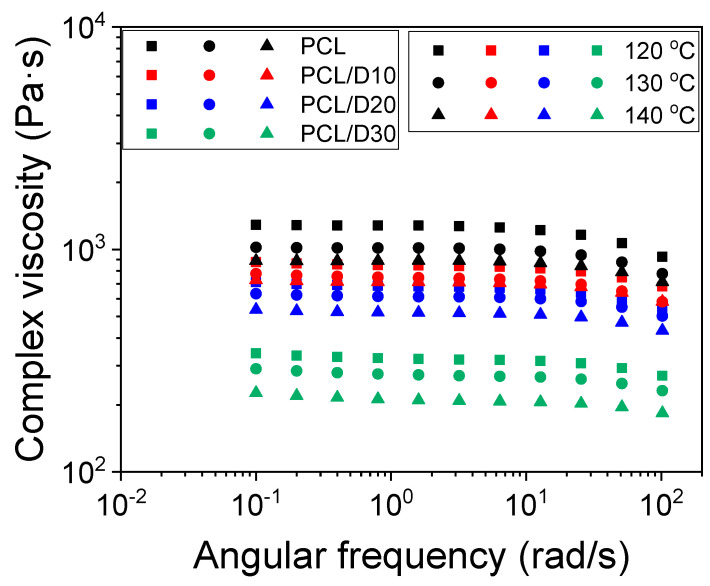
Complex viscosity of pure PCL and PCL-based composites.

The complex viscosity from the rotational rheometer test result was decreased with increasing DMSO_2_ concentration, and the complex viscosity was further decreased with increasing temperature (Figure 2). Moreover, the complex viscosity decreased at high angular frequency regardless of the measuring temperature and material, indicating shear-thinning behavior, in which the viscosity decreased as the angular frequency increased. In polymer materials, the molecular weight of the material strongly influences the complex viscosity, where higher molecular weight polymers tend to exhibit higher viscosity. As a result, the addition of DMSO_2_ in the composites led to lower viscosity due to DMSO_2_ having a much lower molecular weight compared to pure PCL.

The Carreau–Yasuda model is one of the most recognized equations for characterizing pseudoplastic flow across a spectrum of shear rates spanning from zero to infinity [31]. The complex viscosity can be calculated by Equation (1) from the storage modulus and the loss modulus, and then the zero-shear viscosity from the Carreau–Yasuda model can be mathematically represented as follows:

(3) $$\eta^{*}=\eta_{0}{\left[1+{\left(\lambda \omega \right)}^{a}\right]}^{\frac{n-1}{a}},$$

where $\eta_{0}$ signifies the zero-shear viscosity, λ is the relaxation time, n corresponds to the power-law index, and a is a dimensionless parameter. Many researchers have made efforts to analyze the complex viscosity of both polymers and polymer-based composites utilizing this equation [32,33,34]. The calculated zero-shear viscosity and the parameter values of the Carreau–Yasuda model are documented in Table 2. The accuracy of the model in comparison to experimental data was assessed using the coefficient of determination (R^2^). The incorporation of DMSO_2_ into the PCL matrix reduced approximately 33, 46, and 74% at DMSO_2_ concentrations of 10, 20, and 30 wt%, respectively, when the test temperature was at 120 °C.

### 3.2. Material Extrusion Velocity

**Figure 3:**
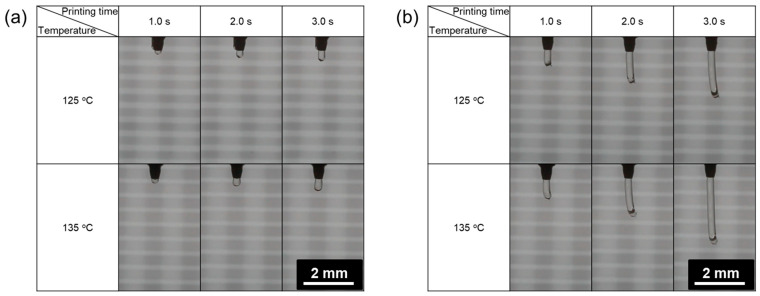
Extruded material length at (**a**) 138 kPa and (**b**) 552 kPa.

**Figure 4:**
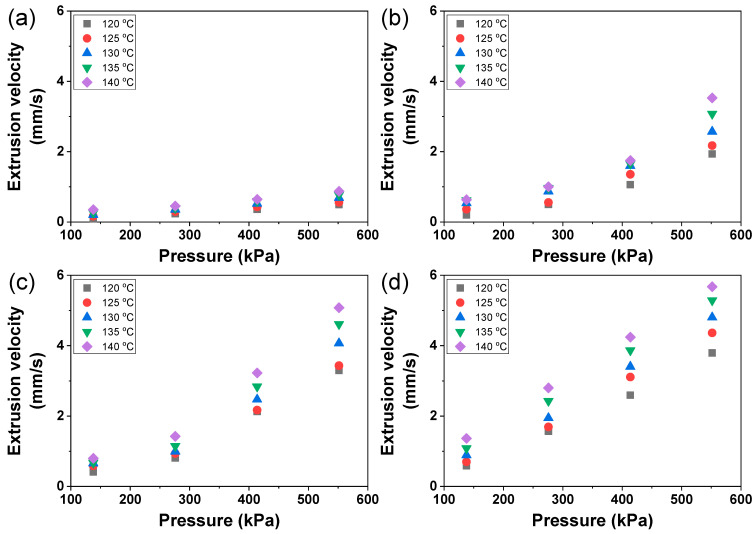
Measured extrusion velocity of (**a**) PCL, (**b**) PCL/D10, (**c**) PCL/D20, and (**d**) PCL/D30.

The extruded material was assumed to form a straight line during printing, and the extrusion velocity was calculated by dividing the extruded material length by the time. According to the observation of the material extrusion, the pressure change seemed to have more impact on the extrusion velocity compared to the change in the temperature (Figure 3). The extrusion velocity was measured in the range of 0.0850–6.58 mm/s throughout all printing conditions and materials. PCL exhibited the narrowest velocity range, while PCL/D30 demonstrated the widest range, as illustrated in Figure 4.

At the printing temperature and pressure of 120 °C and 138 kPa, respectively, the extrusion velocity of PCL was 0.0850 mm/s. The extrusion velocity of PCL increased by a factor of 2.4 and 4.2 when the printing temperature was elevated to 130 °C and 140 °C, respectively, while maintaining the same printing pressure. Similarly, when the printing pressure was set to 552 kPa, the extrusion velocity of PCL at 120 °C was 0.4964 mm/s. With this higher printing pressure (552 kPa), the extrusion velocity increased less, reaching 1.4 and 1.8 times its initial value when the printing temperature was raised to 130 °C and 140 °C, respectively.

For PCL/D30 at a printing pressure of 138 kPa, the extrusion velocity was recorded as 0.5908 mm/s when the printing temperature was 120 °C. As the printing temperature was increased to 130 °C and 140 °C at the same printing pressure, the extrusion velocity of PCL/D30 increased by 1.5 and 2.3 times, respectively. When the printing pressure was elevated to 552 kPa, the extrusion velocity of PCL/D30 at 120 °C of printing temperature reached 3.7899 mm/s. With this higher printing pressure, the extrusion velocity still increased by 1.3 and 1.5 times when the printing temperature was raised to 130 °C and 140 °C, respectively.

The more pronounced change in the extrusion velocity in pure PCL compared to PCL/DMSO_2_ composites can be related to the zero-shear viscosity change of the materials. Section 3.1 shows that the zero-shear viscosity change of pure PCL by temperature was more remarkable than PCL/DMSO_2_ composites, and the extrusion velocity change of pure PCL by temperature showed a noticeable difference. The extrusion velocity of pure PCL and PCL/DMSO_2_ composites can be expressed by the function of the material viscosity. Lee et al. [35] proposed an analytical extrusion velocity (v) equation that depends on the reciprocal of the material viscosity as follows:

(4) $$v=\left(\frac{1}{{\eta_{0}}^{1/m}}\right)\left(\frac{m}{m+1}\right)\left(-\frac{2L_{n}}{\Delta P}\right){\left(-\frac{\Delta P}{2L_{n}}r_{n}-\tau_{0}\right)}^{\frac{m+1}{m}},$$

where m is a constant, $L_{n}$ signifies a nozzle length, ΔP corresponds to the pressure difference between the molten material and ambient pressure, $r_{n}$ denotes the nozzle inner diameter, and $\tau_{0}$ is an activation energy. This equation allowed for the calculation of extrusion velocity changes due to the material viscosity alterations. The calculated results fitted well with the experimental data, exhibiting an absolute error below 0.1 mm/s. The experimental and calculated extrusion velocity values are presented in Table 3.

The extrusion velocity of PCL exhibited more sensitivity to changes in printing temperature when the printing pressure was lower. This phenomenon can be attributed to the difference in pressure between the molten material and ambient pressure. The ambient pressure was generally assumed to remain constant, and the pressure difference between the molten material and ambient pressure primarily depended on changes in the printing pressure. Moreover, the printing pressure had a direct impact on the shear rate during material extrusion, and the viscosity at high shear rates decreased due to the shear-thinning behavior of the material. Consequently, when high-temperature and high-pressure conditions were applied, they resulted in a substantial acceleration of the extrusion velocity.

These results implied that the material viscosity depended on the printing conditions, such as the printing temperature and pressure, resulting in a noticeable change in the extrusion velocity. Therefore, the importance of carefully controlling the printing conditions to achieve the desired extrusion velocity was emphasized.

### 3.3. Printed Strut Diameter

**Figure 5:**
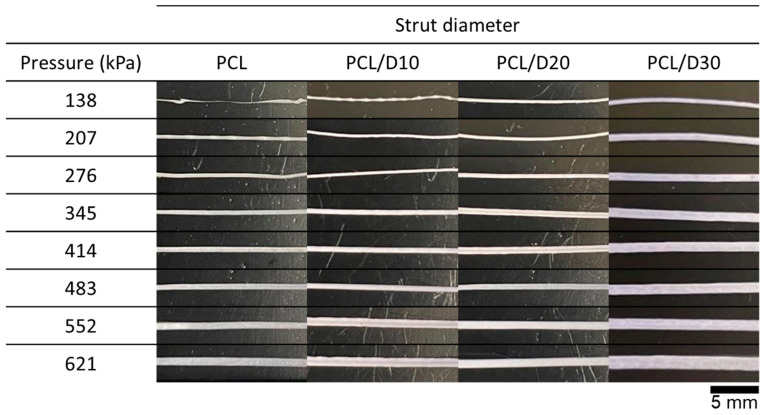
Printed strut of PCL composites at 120 °C.

The strut diameter positively correlated with the pressure for both PCL and PCL/DMSO_2_ composite materials. The one-dimensional printing with 2 mm of length exhibited linear lines under most printing conditions, but PCL at 138 and 207 kPa, as well as PCL/D10 at 138 kPa, displayed a non-linear line when the printing temperature was set as 120 °C (Figure 5).

**Figure 6:**
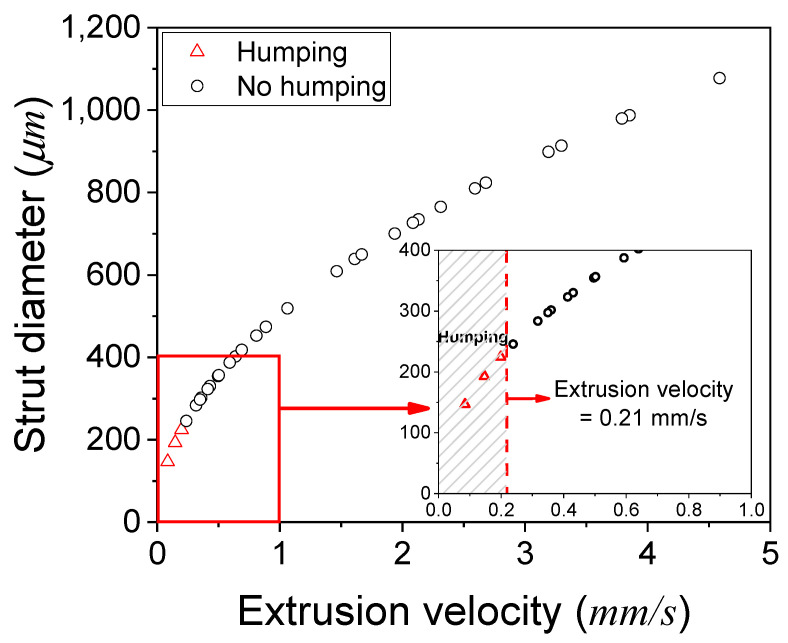
Occurrence of the humping phenomenon in the relationship between the extrusion velocity and strut diameter.

A humping phenomenon similar to occurring in high-speed welding [36] was observed at 138 and 207 kPa of PCL and 138 kPa of PCL/D10 during the line printing test. The pressure conditions directly affected the extrusion velocity, and the humping phenomenon was obviously detected when the extrusion velocity was slow compared to the nozzle moving speed (0.8 mm/s), as shown in Figure 6.

Increasing the extrusion velocity or decreasing the nozzle moving speed can solve the humping phenomenon. By adjusting either the extrusion velocity or the nozzle moving speed, or even a combination of both, it can be feasible to identify the optimal printing parameters that effectively prevent the occurrence of the humping phenomenon.

**Figure 7:**
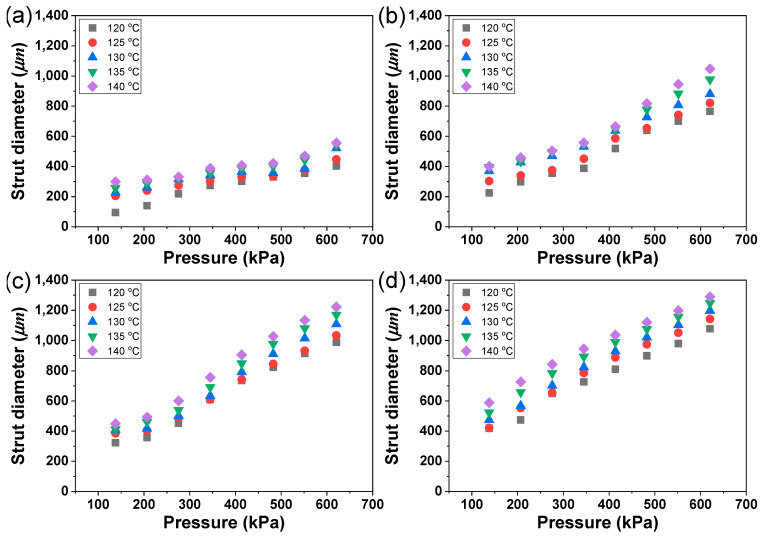
Measured strut diameter of (**a**) PCL, (**b**) PCL/D10, (**c**) PCL/D20, and (**d**) PCL/D30.

The strut diameter was measured on the captured printed line. The minimum strut diameter observed was 147 µm under PCL at 120 °C of printing temperature and 138 kPa of pressure. In contrast, the maximum strut diameter recorded was 1290 µm under PCL/D30 at 140 °C of printing temperature and 621 kPa of pressure (Figure 7). The strut diameter of PCL and PCL/D30 increased by 2.0 and 1.4 times, respectively, with a temperature increase of 20 °C at 138 kPa, while it increased by 1.4 and 1.2 times, respectively, with the same temperature increase at 621 kPa. Similarly, when the pressure increased by 483 kPa, the strut diameter of PCL and PCL/D30 increased by 2.7 and 2.6 times, individually, at 120 °C, while it increased by 1.9 and 2.2 times at 140 °C.

Generally, the strut diameter showed a trend of being thick with the increase in printing temperature, pressure, and DMSO_2_ concentration. This phenomenon can be explained by the volume flow rate of the molten material from the nozzle. A high-volume flow rate showed the capacity to produce a thicker strut diameter, particularly when the total printing time remained constant across all printing conditions. The volume flow rate (Q) can be given by:

(5) $$Q=\int_{0}^{r_{n}}2\pi rvdr,$$

where r represents the inner diameter of the nozzle, which was consistently set at 450 µm throughout this study. This equation for the volume flow rate is recognized as a generalized volumetric flow rate model for non-Newtonian fluids [37]. Therefore, the primary factor influencing the volume flow rate was the extrusion velocity of the molten materials. As explained in Section 3.2, the extrusion velocity was related to the printing temperature, pressure, and DMSO_2_ concentration, and it increased with a decrease in material viscosity under these variable conditions. The rheological behavior of the material affected the extrusion velocity and consequently determined the strut diameter by changing the volume flow rate.

The strut diameter at a specific printing temperature and pressure can be derived from the volumetric flow rate with an assumption that the cross-section of the strut is circular. Xia et al. [38] introduced the equation about the strut diameter ($d_{s}$) as follows:

(6) $$d_{s}=\sqrt{\frac{4Q}{v\pi }}$$

The actual and expected strut diameters, along with their respective absolute errors, are documented in Table 4.

The absolute error concerning the strut diameter displayed an upward trend as the printing temperature elevated. Additionally, an increase in the DMSO_2_ concentration led to a corresponding increase in the absolute error. This outcome can be attributed to the initial assumption that the strut possessed a circular cross-section. However, due to factors such as molten material surface tension and density, the strut cannot maintain a perfectly circular cross-section. Instead, it might resemble an elliptical shape in its cross-section [39]. Therefore, it should need a more complicated equation to predict the strut diameter exactly.

Consequently, the material viscosity change led to a decrease in the extrusion velocity, flow rate, and, ultimately, the strut diameter changes when altering the printing temperatures and DMSO_2_ concentration. These findings were similar to the effect of temperature and pressure on the extrusion velocity. Temperature and pressure increases can directly affect the dimensional characteristics of the printed object. Therefore, it was crucial to carefully control these parameters to achieve the desired strut diameter and ensure dimensional accuracy in the fabrication of the 3D bio-scaffold.

### 3.4. Pore in 3D Scaffold

In this study, the 3D scaffold was fabricated using the material extrusion 3D printing method. The printing process involved layer-by-layer deposition, and the nozzle moving speed was set at 0.8 mm/s. The scaffold design consisted of a cylinder shape with a diameter of 2 mm and a strut pitch of 700 µm. A total of five layers were used in the scaffold construction.

**Figure 8:**
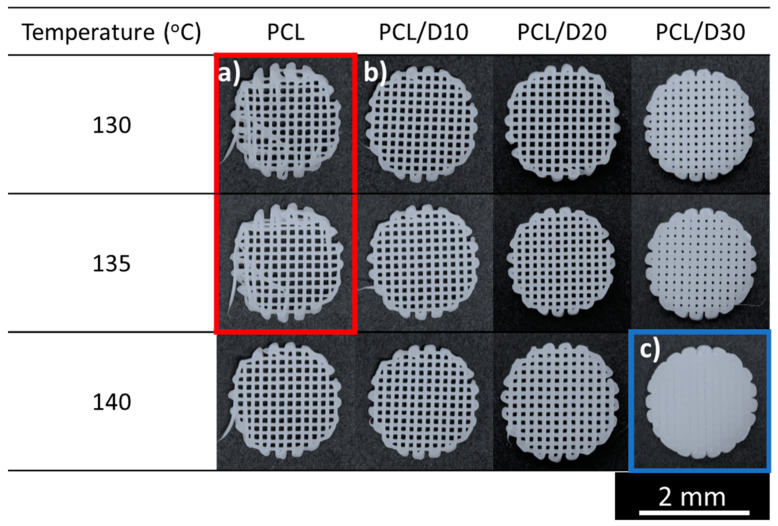
Top view of the printed 3D scaffold under 207 kPa: section (a) irregular-pore zone in the red box, (b) normal-pore zone, and (c) no-pore zone in the blue box.

It was observed that the PCL scaffold at 130 and 135 °C exhibited an irregular pore shape due to the relatively slow extrusion velocity of PCL compared to the nozzle moving speed when the printing pressure was below 207 kPa. The extrusion velocity of the two cases was below 0.21 mm/s. However, the PCL and DMSO_2_ composites were able to form well-shaped scaffolds. On the other hand, PCL/D30 at 140 °C was unable to generate pores because the strut diameter was thicker than the designed strut pitch, which meant that the strut diameter should be thinner than the designed strut pitch (Figure 8).

**Figure 9:**
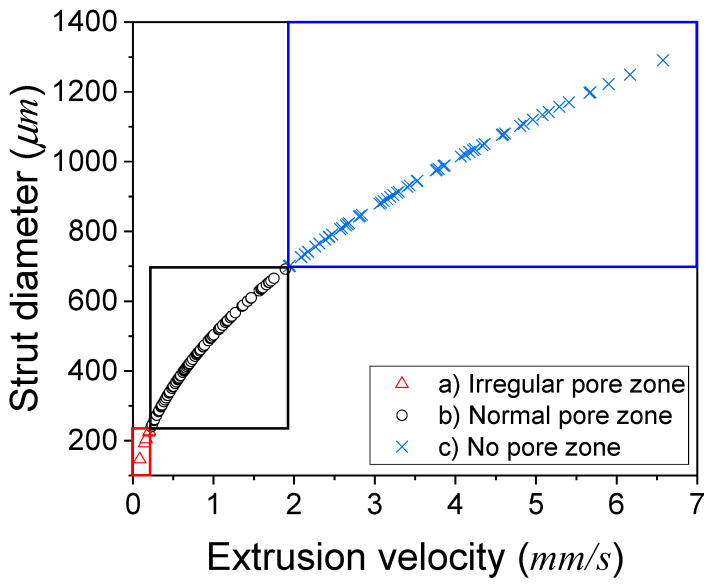
Pore status in the 3D scaffold by the extrusion velocity and strut diameter: section (a) irregular-pore zone in the red box, (b) normal-pore zone in the black box, and (c) no-pore zone in the blue box.

The printed strut shape and diameter determined the pore status in the 3D scaffold, and the strut shape and diameter were settled by the extrusion velocity. The pore status in the 3D scaffold can be expressed by the relationship between the extrusion velocity and strut diameter (Figure 9).

The slow extrusion velocity (<0.21 mm/s) caused the humping phenomenon, resulting in an irregular-pore zone, while the thicker strut diameter than the designed strut pitch caused the no-pore zone. Therefore, the normal-pore zone can be proposed between the irregular-pore zone and no-pore zone.

## 4. Conclusions

The objective of this study was to evaluate the rheological characteristics change of PCL composites by adding DMSO_2_ and study the printing behavior by the rheological characteristics of pure PCL and PCL/DMSO_2_ composites. Rheological properties hold significant importance in the 3D printing process, as they dictate the material flow and associated behavior. Analyzing the rheological properties allows for a comprehensive understanding of the printing behavior, encompassing material extrusion and stacking phenomena.

The rheological properties of the tested materials, including pure PCL and PCL/DMSO_2_ composites, were assessed using a rotational rheometer to characterize the storage modulus, loss modulus, and complex viscosity. At temperatures of 120, 130, and 140 °C, all materials displayed liquid-like behavior, as evidenced by higher loss modulus values compared to the storage modulus. Both the storage modulus and loss modulus exhibited a decreasing trend with rising temperatures and increasing DMSO_2_ concentrations in the PCL matrix. The materials, including DMSO_2_, demonstrated shear-thinning behavior, wherein the viscosity decreased with an increase in angular frequency. Furthermore, the addition of DMSO_2_ into the PCL matrix or elevation of temperature contributed to a reduction in material viscosity. This behavior can be attributed to the dilution effect of DMSO_2_, which possesses lower viscosity in comparison to PCL. The zero-shear viscosity was calculated using the Carreau–Yasuda model. The values by DMSO_2_ concentrations of 10, 20, and 30 wt% were reduced by 33, 46, and 74%, respectively, at 120 °C.

The extrusion velocity of all the tested materials ranged from 0.0850 to 6.58 mm/s. The extrusion velocity was influenced by the reciprocal of viscosity, and PCL exhibited higher sensitivity to temperature changes. As a result, the extrusion velocity of pure PCL exhibited more significant fluctuations, primarily due to the substantial variations in viscosity caused by changes in temperature compared to PCL/DMSO_2_ composites. At extrusion velocities below 0.21 mm/s, the printed lines exhibited an unstable and wavy line, such as a humping phenomenon. Distinct material viscosities resulted in variations in the extrusion velocity, flow rate, and strut diameter during the printing process. Based on the extrusion velocity and strut diameter, the pore shape was categorized into three zones: irregular, normal, and no-pore zone. The irregular-pore zone was observed when the extrusion velocity was insufficient to print a straight line. Conversely, the no-pore zone was determined when the strut diameter exceeded the designed strut pitch.

## Figures and Tables

**Table 1:** Material properties of PCL and DMSO_2_.

Materials	Appearance	Molecular Weight (g/mol)	Density (kg/m^3^)	Melting Temperature (°C)
PCL	Powder	50,000	1145	58–60
DMSO_2_	Powder	94.13	1450	107–109

**Table 2:** The values of zero-shear viscosity and Carreau–Yasuda model parameters.

Materials	Temperature (°C)	$\eta_{0}$ (Pa·s)	λ (s)	n	a	R^2^
PCL	120	1286.6	0.0108	0.54	1.05	0.9998
	130	1018.9	0.0128	0.64	1.14	0.9996
	140	889.5	0.0204	0.77	1.42	0.9995
PCL/D10	120	863.0	0.0017	0.23	0.76	0.9859
	130	762.7	0.0020	0.20	0.78	0.9856
	140	720.1	0.0018	0.18	0.91	0.9941
PCL/D20	120	696.9	0.0021	0.19	0.80	0.9843
	130	622.2	0.0020	0.17	0.87	0.9877
	140	526.9	0.0016	0.18	0.83	0.9845
PCL/D30	120	333.8	0.0006	0.38	0.57	0.9511
	130	286.5	0.0002	0.51	0.44	0.9458
	140	229.1	0.0000	0.72	0.26	0.9220

**Table 3:** The actual and expected values of the extrusion velocity at 138 and 552 kPa of printing pressure.

Materials	Temp. (°C)	Extrusion Velocity at 138 kPa			Extrusion Velocity at 552 kPa		
		Actual (mm/s)	Expected (mm/s)	Error (mm/s)	Actual (mm/s)	Expected (mm/s)	Error (mm/s)
PCL	120	0.0850	0.1298	0.04	0.4964	0.5154	0.02
	130	0.2041	0.1927	0.01	0.6844	0.6578	0.03
	140	0.3532	0.3379	0.02	0.8732	0.7779	0.10
PCL/D10	120	0.1990	0.2125	0.01	1.9373	1.9997	0.06
	130	0.5377	0.5700	0.03	2.5715	2.6211	0.05
	140	0.6336	0.6705	0.04	3.5298	3.4972	0.03
PCL/D20	120	0.4125	0.4510	0.04	3.2969	3.2108	0.09
	130	0.6584	0.5871	0.07	4.0675	4.1300	0.06
	140	0.7997	0.7455	0.05	5.0799	4.9956	0.08
PCL/D30	120	0.5908	0.6526	0.06	3.7899	3.8229	0.03
	130	0.8885	0.9563	0.07	4.8039	4.8857	0.08
	140	1.3662	1.3946	0.03	5.6769	5.7564	0.08

**Table 4:** The actual and expected values of the strut diameter at 138 and 621 kPa of printing pressure.

Materials	Temp. (°C)	Strut Diameter at 138 kPa			Strut Diameter at 621 kPa		
		Actual (µm)	Expected (µm)	Error (µm)	Actual (µm)	Expected (µm)	Error (µm)
PCL	120	146.6	141.3	5.4	402.2	396.1	6.1
	130	227.3	220.8	6.5	520.8	510.7	10.0
	140	299.0	292.5	6.5	556.7	542.2	14.4
PCL/D10	120	224.4	216.9	7.5	765.1	775.4	10.3
	130	368.9	359.8	9.1	879.8	866.9	12.9
	140	400.5	387.0	13.5	1047.3	1028.3	19.0
PCL/D20	120	323.1	312.9	10.3	987.4	972.8	14.6
	130	408.2	390.5	17.7	1107.9	1089.2	18.7
	140	449.9	429.4	20.5	1222.3	1198.2	24.1
PCL/D30	120	418.2	401.4	16.7	1077.5	1058.6	18.9
	130	474.2	452.0	22.2	1197.3	1172.1	25.3
	140	588.1	564.1	23.9	1290.2	1262.1	28.0

## Data Availability

The original contributions presented in the study are included in the article, further inquiries can be directed to the corresponding author.

## References

[1] Jang J.-W., Min K.-E., Kim C., Shin J., Lee J., Yi S. (2023). Scaffold Characteristics, Fabrication Methods, and Biomaterials for the Bone Tissue Engineering. Int. J. Precis. Eng. Manuf..

[2] Swinehart I.T., Badylak S.F. (2016). Extracellular matrix bioscaffolds in tissue remodeling and morphogenesis. Dev. Dyn..

[3] Min K.-E., Jang J.-W., Shin J., Kim C., Yi S. (2023). Development of Prediction Method for Dimensional Stability of 3D-Printed Objects. Appl. Sci..

[4] Udofia E.N., Zhou W. Microextrusion based 3D printing–a review. Proceedings of the 2018 International Solid Freeform Fabrication Symposium.

[5] Yang E., Miao S., Zhong J., Zhang Z., Mills D.K., Zhang L.G. (2018). Bio-based polymers for 3D printing of bioscaffolds. Polym. Rev..

[6] Raghunath J., Rollo J., Sales K.M., Butler P.E., Seifalian A.M. (2007). Biomaterials and scaffold design: Key to tissue-engineering cartilage. Biotechnol. Appl. Biochem..

[7] Hutmacher D.W. (2000). Scaffolds in tissue engineering bone and cartilage. Biomaterials.

[8] MacNeil S. (2007). Progress and opportunities for tissue-engineered skin. Nature.

[9] Nerem R.M., Seliktar D. (2001). Vascular tissue engineering. Annu. Rev. Biomed. Eng..

[10] Ng W.L., An J., Chua C.K. (2024). Process, material, and regulatory considerations for 3D printed medical devices and tissue constructs. Engineering.

[11] Wales D.J., Keshavarz M., Howe C., Yeatman E. (2022). 3D Printability Assessment of Poly (octamethylene maleate (anhydride) citrate) and Poly (ethylene glycol) Diacrylate Copolymers for Biomedical Applications. ACS Appl. Polym. Mater..

[12] Duty C., Ajinjeru C., Kishore V., Compton B., Hmeidat N., Chen X., Liu P., Hassen A.A., Lindahl J., Kunc V. (2018). What makes a material printable? A viscoelastic model for extrusion-based 3D printing of polymers. J. Manuf. Process..

[13] Gosset A., Barreiro-Villaverde D., Becerra Permuy J.C., Lema M., Ares-Pernas A., Abad López M.J. (2020). Experimental and numerical investigation of the extrusion and deposition process of a poly (lactic acid) strand with fused deposition modeling. Polymers.

[14] Kim H.C., Panicker P.S., Muthoka R.M., Jang J., Yi S., Kim J. (2022). A study in bio-nanocomposite based on polycaprolactone reinforced by cellulose nanocrystal. Proceedings of the Nano-, Bio-, Info-Tech Sensors, and Wearable Systems 2022.

[15] Kyle S., Jessop Z.M., Al-Sabah A., Whitaker I.S. (2017). ‘Printability’ of candidate biomaterials for extrusion based 3D printing: State-of-the-art. Adv. Healthc. Mater..

[16] Vyas C., Zhang J., Øvrebø Ø., Huang B., Roberts I., Setty M., Allardyce B., Haugen H., Rajkhowa R., Bartolo P. (2021). 3D printing of silk microparticle reinforced polycaprolactone scaffolds for tissue engineering applications. Mater. Sci. Eng. C.

[17] Vurat M., Parmaksiz M. (2021). Mechanical Evaluation of 3D Printed Polycaprolactone Scaffolds: Effect of Molecular Weight. Int. J. 3D Print. Technol. Digit. Ind..

[18] Siddiqui N., Asawa S., Birru B., Baadhe R., Rao S. (2018). PCL-Based Composite Scaffold Matrices for Tissue Engineering Applications. Mol. Biotechnol..

[19] Bakhsheshi-Rad H.R., Hamzah E., Ying W.S., Razzaghi M., Sharif S., Ismail A.F., Berto F. (2021). Improved bacteriostatic and anticorrosion effects of polycaprolactone/chitosan coated magnesium via incorporation of zinc oxide. Materials.

[20] Murugan S., Parcha S.R. (2021). Fabrication techniques involved in developing the composite scaffolds PCL/HA nanoparticles for bone tissue engineering applications. J. Mater. Sci. Mater. Med..

[21] Wang F., Tankus E.B., Santarella F., Rohr N., Sharma N., Märtin S., Michalscheck M., Maintz M., Cao S., Thieringer F.M. (2022). Fabrication and characterization of PCL/HA filament as a 3D printing material using thermal extrusion technology for bone tissue engineering. Polymers.

[22] Noroozi N., Thomson J.A., Noroozi N., Schafer L.L., Hatzikiriakos S.G. (2012). Viscoelastic behaviour and flow instabilities of biodegradable poly (ε-caprolactone) polyesters. Rheol. Acta.

[23] Arraiza A.L., Sarasua J., Verdu J., Colin X. (2007). Rheological behavior and modeling of thermal degradation of poly (∊-caprolactone) and poly (L-lactide). Int. Polym. Process..

[24] Germiniani L.G.L., da Silva L.C.E., Plivelic T.S., Gonçalves M.C. (2018). Poly(ε-caprolactone)/cellulose nanocrystal nanocomposite mechanical reinforcement and morphology: The role of nanocrystal pre-dispersion. J. Mater. Sci..

[25] Doyle S.E., Henry L., McGennisken E., Onofrillo C., Bella C.D., Duchi S., O’Connell C.D., Pirogova E. (2021). Characterization of Polycaprolactone Nanohydroxyapatite Composites with Tunable Degradability Suitable for Indirect Printing. Polymers.

[26] Jang J.-W., Min K.-E., Kim C., Wern C., Yi S. (2023). PCL and DMSO_2_ Composites for Bio-Scaffold Materials. Materials.

[27] Min K.-E., Jang J.-W., Yi S., Kim C. (2023). Role of binder on yield strength of polycaprolactone/dimethylsulfone composites for bio-applications. J. Mater. Res. Technol..

[28] Backes E.H., Harb S.V., Beatrice C.A.G., Shimomura K.M.B., Passador F.R., Costa L.C., Pessan L.A. (2022). Polycaprolactone usage in additive manufacturing strategies for tissue engineering applications: A review. J. Biomed. Mater. Res. Part B Appl. Biomater..

[29] Lertwimolnun W., Vergnes B. (2005). Influence of compatibilizer and processing conditions on the dispersion of nanoclay in a polypropylene matrix. Polymer.

[30] Máca J., Vondrák J., Sedlaříková M. (2014). Use of Dimethyl Sulfone as Additive in Aprotic Electrolytes. ECS Trans..

[31] Zare Y., Park S.P., Rhee K.Y. (2019). Analysis of complex viscosity and shear thinning behavior in poly (lactic acid)/poly (ethylene oxide)/carbon nanotubes biosensor based on Carreau–Yasuda model. Results Phys..

[32] Aho J., Syrjälä S. (2010). Measurement of the pressure dependence of viscosity of polymer melts using a back pressure-regulated capillary rheometer. J. Appl. Polym. Sci..

[33] Boyd J., Buick J.M., Green S. (2007). Analysis of the Casson and Carreau-Yasuda non-Newtonian blood models in steady and oscillatory flows using the lattice Boltzmann method. Phys. Fluids.

[34] Javed M.A., Ali N., Arshad S., Shamshad S. (2021). Numerical approach for the calendering process using Carreau-Yasuda fluid model. J. Plast. Film Sheeting.

[35] Lee J., Walker J., Natarajan S., Yi S. (2019). Prediction of geometric characteristics in polycaprolactone (PCL) scaffolds produced by extrusion-based additive manufacturing technique for tissue engineering. Rapid Prototyp. J..

[36] Soderstrom E., Mendez P. (2006). Humping mechanisms present in high speed welding. Sci. Technol. Weld. Join..

[37] Luo Y., Peden J. (1990). Flow of non-Newtonian fluids through eccentric annuli. SPE Prod. Eng..

[38] Xia H., Lu J., Dabiri S., Tryggvason G. (2018). Fully resolved numerical simulations of fused deposition modeling. Part I: Fluid flow. Rapid Prototyp. J..

[39] Comminal R., Jafarzadeh S., Serdeczny M., Spangenberg J. (2021). Estimations of Interlayer Contacts in Extrusion Additive Manufacturing Using a CFD Model. Proceedings of the International Conference on Additive Manufacturing in Products and Applications.

